# Autism and Intellectual Disability Associated with Mitochondrial Disease and Hyperlactacidemia

**DOI:** 10.3390/ijms16023870

**Published:** 2015-02-11

**Authors:** José Guevara-Campos, Lucía González-Guevara, Omar Cauli

**Affiliations:** 1 “Felipe Guevara Rojas” Hospital, Pediatrics Service, University of Oriente, El Tigre-Anzoátegui, 6034 Venezuela, Spain; E-Mail: joguevara90@hotmail.com; 2 “Felipe Guevara Rojas” Hospital, Epilepsy and Encephalography Unit, El Tigre-Anzoátegui, 6034 Venezuela, Spain; E-Mail: proyectoasociacion@hotmail.com; 3 Department of Nursing, University of Valencia, 46010 Valencia, Spain

**Keywords:** autism, possible mitochondrial disease, vitamins, intellectual disability, muscular tone

## Abstract

Autism spectrum disorder (ASD) with intellectual disability (ID) is a life-long debilitating condition, which is characterized by cognitive function impairment and other neurological signs. Children with ASD-ID typically attain motor skills with a significant delay. A sub-group of ASD-IDs has been linked to hyperlactacidemia and alterations in mitochondrial respiratory chain activity. The objective of this report is to describe the clinical features of patients with these comorbidities in order to shed light on difficult diagnostic and therapeutic approaches in such patients. We reported the different clinical features of children with ID associated with hyperlactacidemia and deficiencies in mitochondrial respiratory chain complex II–IV activity whose clinical presentations are commonly associated with the classic spectrum of mitochondrial diseases. We concluded that patients with ASD and ID presenting with persistent hyperlactacidemia should be evaluated for mitochondrial disorders. Administration of carnitine, coenzyme Q10, and folic acid is partially beneficial, although more studies are needed to assess the efficacy of this vitamin/cofactor treatment combination.

## 1. Introduction

Intellectual disability (ID) represents a significant social and economic burden; About 3% of the Western population is diagnosed with ID, and these patients require lifelong care [1,2,3]. An individual is considered to have an ID based on the following three criteria: (1) An intellectual functioning level (IQ) below 70; (2) Significant limitations exist in two or more adaptive skill areas (communication, self-care, home living, social skills, leisure, health and safety, self-direction, functional academics, community use, and work); and (3) the condition manifests itself before the age of 18 [4,5]. ID is sometimes associated with autism spectrum disorders (ASDs), behavioral disorders (hyperactivity, irritability, and self-injurious behavior), epilepsy, and/or other neurological disabilities (ataxia, hypotonia, sensorial alterations), all resulting in psychological, social, and economic burdens [1,6,7]. There are also some genetic causes of ID, which constitute 30%–50% of cases [3,7,8,9], however in the majority of instances the etiology is unknown. Assessment of molecular defects that result in synaptic deficits as well as defining suitable therapeutic treatments for these deficits remains a challenge [10].

Mitochondrial diseases can develop because of mitochondrial (mtDNA) or nuclear DNA (nDNA) mutations, however in many cases no mutations are found. These mutations are uncommon in the general population and have an estimated prevalence of around 1:8500 [11]. The extremely heterogeneous clinical presentation of mtDNA and nDNA mutations often causes diagnostic difficulties and thus many patients are diagnosed with mitochondrial disease without identifying the mtDNA or nDNA mutation responsible for the ASD-ID [2]. Additionally, the same genetic mutation can give rise to multiple varied phenotypes [12]. Mitochondrial respiratory chain (electron transport chain; ETC) disorders can be difficult to recognize clinically because of their diverse symptoms and clinical presentations and, as we report here, the diagnosis is often reached late in childhood.

Histopathological, biochemical, and genetic findings [13,14] are normally used to aid final diagnosis of possible, probable, or definite mitochondrial disease. The clinical presentation almost always widely varies, making definitive diagnosis of these mitochondrial disorders challenging [15] and although various criteria and checklists have been established these are more reflective of adult disease [13,14,16]. Patients with mitochondrial disease often show signs and symptoms such as heart, pancreas, or liver dysfunction, growth retardation, and fatigability, but sometimes the semiology is different from classical mitochondrial diseases and patients show symptoms associated with neurological deficits such as ID, ASD, abnormal muscle tone, seizures, extrapyramidal movements, and autonomic and ocular dysfunction [17,18].

Here, we report the interesting cases of three patients diagnosed with developmental delay, ID and ASD, and also with a possible mitochondrial disease accompanied by an ETC deficiency accompanied by hyperlactacidemia [14]. These cases have an unusual clinical presentation, which would not normally lead to suspicion of mitochondrial disease. For instance, fewer than three organ systems were involved, and red-flags for neurological involvement *i.e.*, cerebral stroke-like lesions in a nonvascular pattern, basal ganglia disease, recurrent encephalopathy, neurodegeneration, epilepsia partialis continua, myoclonus, ataxia, magnetic resonance imaging (MRI) findings consistent with Leigh disease, *etc.*, were not present [2]. We provide useful clinical evidence that patients presenting only ID and an alteration in muscular tone but in the absence of any other systemic signs may, according to the current classification of these disorders, also have a mitochondrial ETC disorder [14]. Children with ID and ASD who show no systemic signs should therefore be evaluated for comorbid mitochondrial disorders. In addition we describe some beneficial effects of combined pharmacological treatment with antipsychotic drugs and vitamins.

## 2. Results

### 2.1. Medical History and Clinical Examination

**Patient 1:** A 17-month-old boy was referred to the neuropediatrician because of generalized hypotonia and reduced cranial growth perimeter. He was the full-term product of a third pregnancy (preceded by a spontaneous miscarriage) resulting from a non-consanguineous relationship. His birth weight was 4.2 kg (60th percentile) and height was 51 cm (50th percentile), he had normal immunization and had an Apgar score of 9. However, he showed global developmental delay according to the Peabody Developmental Motor Scale 2 (PDMS-2) and the Battelle developmental inventory 2 (BDI-2; see Section 4.1.).

Head support was achieved at seven months, with stable seating and autonomous position attained at 12 months; he started to walk autonomously at 30 months, and his first raw words appeared at 12 months. There was no family history of metabolic or neurological disease. Upon physical examination, his weight was 10 kg (50–75th percentile), height was 72 cm (90th percentile), head circumference was 41.5 cm (10th percentile, microcephaly), and his general condition was regular except for generalized hypotonia and loss of strength in the upper part of his lower-limbs but with normal deep tendon I/IV reflexes. No dysmorphic features, skin blemishes, or cardiopulmonary alterations were observed.

**Patient 2:** A two-year-old girl was referred to the neuropediatrician for psychomotor delay and generalized hypotonia. She was the term product of a first pregnancy resulting from a non-consanguineous relationship. Her birth weight was 2.7 kg (10th percentile), her height was 50 cm (50th percentile), and she had normal immunization. At birth, her Apgar score was 7 because of a delayed birth cry. Head support was achieved at 12 months, with a stable seating position achieved for the first time at 24 months, and autonomous walking at 30 months. Her first raw words appeared at 12 months but she did not produce complete sentences. She exhibited global developmental delay according to the PDMS-2 scale and BDI-2 inventory (see Section 4.1.).

**Figure 1:**
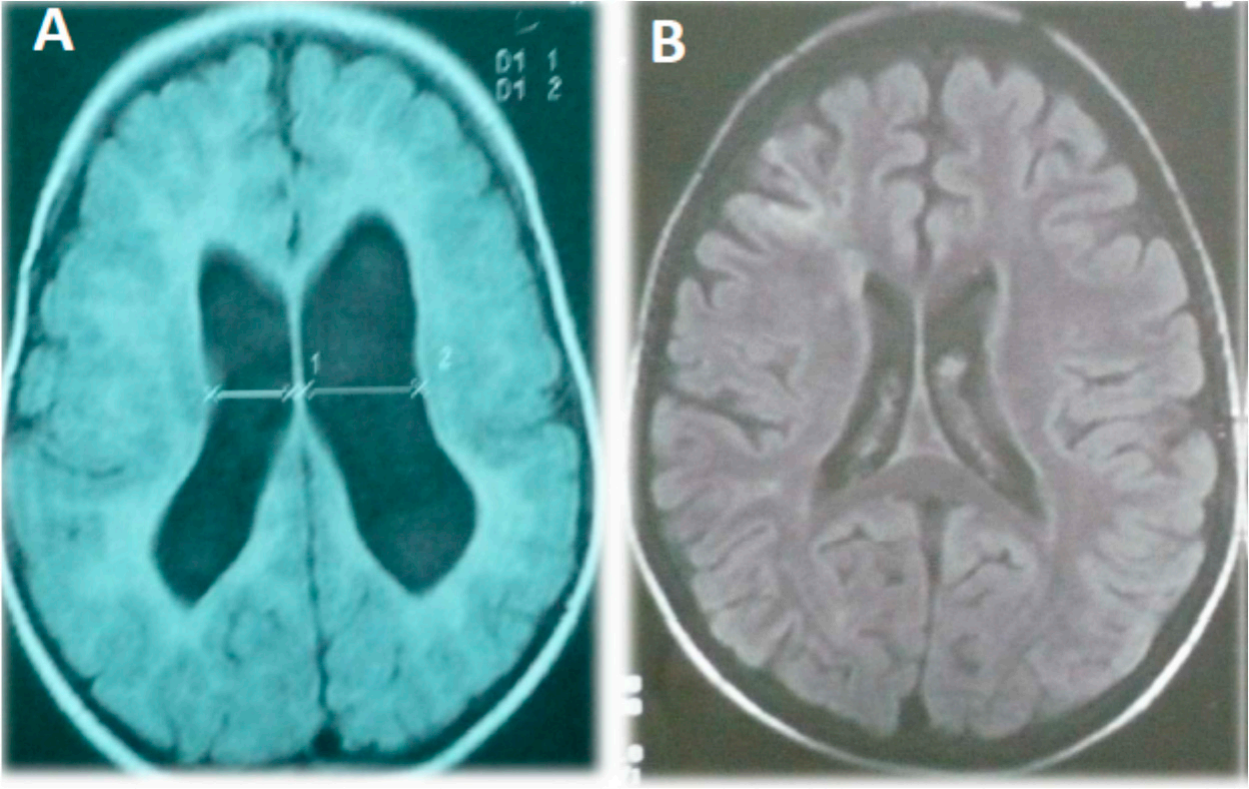
Magnetic resonance image of patient 2 (**A**) and 3 (**B**) showing an increase in lateral cerebral ventricle volume, diffuse enlargement of the cortical spaces, and cortical atrophy.

There was a family history of diabetes (four aunts and her maternal grandfather). Upon physical examination she had a weight of 13.5 kg (50–75th percentile), a height of 93 cm (90th percentile), head circumference of 43 cm (10th percentile, microcephaly), hypertonia in the left hemibody, and deep tendon hyperreflexia in her left-hand side limbs. There were no dysmorphic features, skin blemishes, or pulmonary alterations, and although her mitral valve was hyperelastic it was competent. Her thorax radiography, karyotype, and the EEG were normal, but an MRI showed ventriculomegaly with a prominent cysterna magna but without signs of displacement or compression (Figure 1A). Her associated clinical features are summarized in Table 1.

**Table 1:** Summary of the clinical features of the three patients described in this case study.

ID	Patient 1	Patient 2	Patient 3
	Severe	Moderate	Severe
**Hypotonia**	YES	YES (hypertonia at 1.5 months in the left hemibody)	YES
**EEG Alteration**	NO	NO	YES
**Seizure**	NO	NO	NO
**Developmental Delay (Before age 5)**	YES	YES	YES
**Behavioral Problem (Irritability)**	YES	NO	YES
**PDD-NOS (According to DMS-IV)**	YES (fulfilled 2 criteria of ASD diagnosis: Qualitative abnormalities in communication and reciprocal social interaction)	YES (fulfilled 1 criterion of ASD diagnosis: Qualitative abnormalities in communication)	YES (fulfilled 2 criteria of ASD diagnosis: Qualitative abnormalities in communication and reciprocal social interaction)
**Microcephaly**	YES	YES	NO
**MRI Alteration (Ventricle enlargement)**	YES	YES	YES

ID: Intellectual disability; EEG: Electroencephalography; PDD-NOS: Pervasive developmental disorder-not otherwise specified; DMS-IV: Diagnostic and statistical manual of mental disorders, fourth edition; and MRI: Magnetic resonance imaging.

**Patient 3:** A 19-month-old boy was referred to the neuropediatrician for psychomotor delay because he was unable to maintain an autonomous seating position or to autonomously walk, and he made poor visual contact and could not pronounce any words. He was the term product of a first pregnancy resulting from a non-consanguineous relationship, and there was no family history of metabolic or neurological diseases. His birth weight was 2.9 kg (50th percentile), his height was 74 cm (50th percentile), he received normal immunization, and his Apgar score was 9. He started to autonomously stand up from the age of three and there was autonomous ambulation at 40 months. His first raw words appeared at 12 months but he did not communicate intentionally or make any sentences. He exhibited global developmental delay according to the PDMS-2 scale and BDI-2 inventory (see Section 4.1.), which was later defined as severe ID.

Upon physical examination his weight was 15 kg (50–75th percentile), height was 83 cm (90th percentile), and he had a normal head circumference of 47 cm. He presented moderate generalized hypotonia accompanied by a reduction of strength in his upper limbs and hyperreflexia (deep tendon II/IV reflexes). There were no dysmorphic features, skin blemishes, visceromegaly, or cardiopulmonary alterations. His thorax radiography and karyotype were normal, but a brain MRI showed cerebral atrophy, prominent cysterna magna, and a moderately increased ventricular volume (Figure 1B). An EEG during natural sleep showed generalized spikes of slow waves (1–2 Hz high voltage), although he never presented seizures. He was diagnosed with severe psychomotor delay, ID, and a possible mitochondrial disease due to decreased mitochondrial respiratory chain complex I activity. His clinical features are summarized in Table 1.

### 2.2. Blood and Muscle Laboratory Tests

Blood laboratory test results were normal for hemoglobin, hematocrit, leukocytes, platelets, glucose, urea, creatinine, alkaline phosphatase, alanine and aspartate transaminases (AST and ALT, respectively), total bilirubin concentration, cholesterol (above 145 mg/dL), ammonia, thyroid hormones, and thyrotropin for all three patients. The concentration of organic acids in urine, biotinidase, and cystine/homocysteine, acylcarnitine panel as well as all amino acids (including alanine) were also within the normal range. Due to persistent hyperlactacidemia (analysis was repeated three times on different days) and the clinical phenotypes of all three patients, femoral muscle biopsies were taken to evaluate any metabolic or histological alterations suggestive of a mitochondrial disorders; we measured mitochondrial ETC complex activity at three years for patient 1, 3.5 years for patient 2, and at 2.4 years for patient 3.

**Patient 1:** Blood gas analysis showed metabolic acidosis with pH 7.20, partial pressure of carbon dioxide (PCO_2_) was 50 mmHg, partial pressure of oxygen (PO_2_) was 134 mmHg, bicarbonate (HCO_3_^−^) was 17.6 mEq/L, and the base excess (BE) was −6.8 mEq/L. Serum electrolytes were normal average (sodium, potassium, and chlorine were 139, 4.80 and 105 mEq/L, respectively). Further tests revealed elevated levels of fasting lactate (2.96 mM; normal range 0.95–2.30), postprandial lactate (3.84 mM; normal range 1.95–3.0), fasting pyruvate (0.1 mM; normal range 0.05–0.09), postprandial pyruvate (0.15 mM; normal range 0.08–0.13), fasting lactate pyruvate ratio (29.6), and postprandial lactate pyruvate ratio (25.6). These details are summarized in Table 2.

Analysis of the muscle homogenate revealed a 30%–50% reduction in the enzymatic complexes II, III, and IV, and a slight reduction (12%) in coenzyme Q10 concentration (CoQ10). Mild and non-specific histological alterations in muscle fiber size were observed by optical microscopy. No known mtDNA mutations were found. The final diagnosis was severe ID (35th IQ percentile at age five), a possible mitochondrial disease (based on current diagnostic criteria [13,14]), a pervasive developmental disorder not otherwise specified (according to the DMS-IV criteria and the Autism Diagnostic Interview—Revised), and microcephaly.

**Table 2:** Metabolic energy study performed on muscle biopsy samples. The enzymatic activity of mitochondrial respiratory chain complexes (I–IV) was corrected for the presence of citrate synthase (CS). Succinate dehydrogenase activity was assessed in the presence of phenazine methosulfate, which acts as part of the electron transfer system. The measurement unit for each enzyme/complex activity was mU/U, and this was normalized to CS activity. Lactate values and the lactate/pyruvate ratio found in the plasma are shown. Values that are underlined represent those that are outside the normal range for children without mitochondrial disease.

Enzyme Activity or Metabolite Concentration	Patient 1	Patient 2	Patient 3	Control Values
**NADH: Cit C oxidoreductase (Complex I + III) (mU/U CS)**	**156**	**310**	**253**	**107–560**
**Succinate: Cit C oxidoreductase (Complex II + III) (mU/U CS)**	**49**	**111**	**35**	**75–149**
**Succinate: DCPIP oxidoreductase (Complex II) (mU/U CS)**	**29**	**48**	**27**	**33–69**
**Succinate Dehydrogenase (mU/U CS)**	**74**	**119**	**79**	**57–239**
**Decylubiquinone: Cytochrome C oxidoreductase (Complex III) (mU/U CS)**	**498**	**597**	**615**	**610–1760**
**Cytochrome C oxidase (Complex IV) (mU/U CS)**	**287**	**501**	**291**	**590–1300**
**Coenzyme Q10 (nmol/U CS)**	**2.3**	**2.6**	**2.9**	**2.6–8.4**
**Citrate Synthase (nmol/min/mg)**	**243.7**	**125.7**	**350**	**71–200**
**Fasting Lactate (mM)**	**2.96**	**2.64**	**4.56**	**<2.30**
**Postprandial Lactate (mM)**	**3.84**	**2.82**	**5.15**	**<3**
**Fasting lactate/pyruvate ratio**	**29.6**	**47.7**	**14.2**	**10–15**
**Postprandial lactate/pyruvate ratio**	**25.6**	**42.2**	**24.5**	**10–15**

**Patient 2:** Blood gas analysis showed the following: pH 7.35, PCO_2_: 26.8 mmHg, PO_2_: 98.9 mmHg, HCO_3_: 24.3 mEq/L, and BE: −0.3 mEq/L (as shown in Table 2). Sodium, potassium, and chlorine serum electrolytes were 136, 4.93, and 102 mEq/L, respectively. Fasting lactate was elevated at 2.64 mM, while fasting pyruvate and the fasting lactate pyruvate ratio (0.06 mM and 47.67, respectively, were within the normal range). No histological alterations were observed by optical microscopy. Analysis of the muscle homogenate revealed a significant reduction in the activity of the mitochondrial respiratory chain enzymatic complexes III (decylubiquinol cytochrome c oxidoreductase) and IV (cytochrome c oxidase) to within 5% and 15% of the lower limit of their normal ranges, respectively (Table 2). No known mtDNA mutations were found and the final diagnosis was moderate ID (50th IQ percentile at age five), microcephaly, a pervasive developmental disorder not otherwise specified (using DMS-IV and the criteria of Autism Diagnostic Interview—Revised), and based on current diagnostic criteria [13,14], a possible mitochondrial disease.

**Patient 3:** Blood gas analysis showed metabolic acidosis with pH 7.33, PCO_2_ of 25 mmHg, PO_2_ of 90 mmHg, HCO_3_ at 17.5 mEq/L, and a BE of −5.5 mEq/L. Serum electrolytes were: sodium 144 mEq/L, potassium: 4.7 mEq/L, and chlorine: 101 mEq/L. Other tests revealed significantly elevated fasting lactate (4.56 mM; normal range 0.95–2.30), postprandial lactate (5.15 mM; normal range 1.95–3.0), and fasting pyruvate (0.21 mM; normal range 0.08–0.10). Postprandial pyruvate was slightly above normal (0.15 mM; normal range 0.08–0.13), and fasting lactate pyruvate and postprandial lactate/pyruvate ratios were normal (14.2 and 24.5, respectively). Analysis of the muscle homogenate revealed a significant reduction (by 20%–45%) in the activity of mitochondrial respiratory chain complexes II and IV/I as well as increased CS. These data are summarized in Table 2. Optical microscopy revealed mild and non-specific histological variations in muscular fiber size. No known mtDNA mutations were found. The final diagnosis was moderate ID (55th IQ percentile at age five), a pervasive developmental disorder not otherwise specified (defined by the DMS IV and the criteria of Autism Diagnostic Interview—Revised), and a possible mitochondrial disease based on current diagnostic criteria [13,14].

### 2.3. Pharmacological Treatment

All three patients were started on the same initial treatment regime of 50 mg/Kg/day l-carnitine, a vitamin B complex (50 mg each of vitamin B1and B2, 15 mg B3, 2 mg B6, and 10 mg B12), and 30 mg CoQ10 divided twice daily, and 5 mg folic acid given once a day.

**Patient 1:** After one year of pharmacological treatment his intellectual abilities improved and he reached the 45th IQ percentile. This patient is currently seven years old and does not show any motor delay, motor alterations, or hypotonia. He recently displayed a behavioral disorder characterized by hyperactivity and irritability (as defined by the fifth edition of the diagnostic and statistical manual of mental disorders [DSM-5] by the American psychiatric association [19]), which was accompanied by psychotic reactions. These symptoms, as well as a sleep problem, greatly improved after he was started on risperidone (0.5 mg twice a day). It is unlikely that this antipsychotic drug caused the improvement of his ID because these changes were present before risperidone therapy, rather it is more likely that risperidone allowed him to be more responsive to other therapeutic interventions, and gave him a better ability to demonstrate his cognitive status when assessed. His intellectual abilities still remain stable (an additional year after the implementation of initial pharmacological treatment), however, he still uses very few words and is not able to construct complete sentences; he attends a special school to which he has adapted well.

**Patient 2:** This patient is currently nine years old and she attends a special school; She now walks autonomously with mild ataxia and instability. Once she started pharmacological treatment both signs (ataxia and tremor) disappeared, if CoQ10 is withdrawn she becomes ataxic and a strong hand tremor ensues. She is able to construct simple sentences and to communicate verbally; she understands verbal instructions and presents moderate ID that improved after treatment, reaching the 60th IQ percentile after 10 months of pharmacological treatment. After an additional year of this treatment her ID remains stable and although her hypertonia improved it still remains present at the lower limb level. This patient has never been referred for behavioral or sleep problems.

**Patient 3:** This patient is currently six years old and he attends a special school, which he has accepted well. His language is very poor and he displays episodes of irritability that improved following administration of 0.75 mg risperidone twice a day. Similar to patient 1, improvement of cognitive status was not due to risperidone treatment since it was started eight months after starting the mitochondrial drug combination treatment. ID also improved by 10 IQ points with this pharmacological treatment compared to before starting treatment (he reached the 60th IQ percentile, compared to the 50th measured a year before treatment started).

## 3. Discussion

Mitochondrial respiratory chain complex disorders are the most prevalent group of inherited neurometabolic diseases [2,3]. Classically, their clinical manifestation includes central and peripheral neurological manifestations, usually associated with the involvement of other organs including the eye, heart, liver, and kidneys, and may cause diabetes mellitus and/or sensorineural deafness. Patients with ID and a mitochondrial disease generally present several symptoms which are rarely observed in idiopathic ID, which can include tremor, ataxia, pancreatic or liver dysfunction, cardiac or hematological alterations, growth retardation, and/or ophthalmological or auditory signs [16,20,21,22]. Mitochondrial diseases are caused by abnormalities in the mitochondrial ETC, and may be accompanied by ID and an ASD in some individuals. Hypotonia with a long delay in motor development milestones such as head support, or stable autonomous seating and walking, can also suggest the presence of a mitochondrial disorder.

All three patients described in this study fulfilled the criteria for global developmental delay (under age five) and ID (over age five) according to the consensual terminology [23]. Their cranial MRIs showed a symmetrical increase in lateral cerebral ventricle volume and there was cortical atrophy in case 3. Interestingly, in addition to general hypotonia on the right side of her body, patient 2 also showed additional spasticity on the left side. This clinical finding probably represents a prior insult to the right hemisphere, as shown by asymmetry of lateral ventricles in her MRI image. Non-specific global MRI abnormalities such as abnormal or delayed myelination, atrophy, ventricle enlargement, or specific brain structural changes are common in patients with mitochondrial disorders and/or ID, especially those with clinical central nervous system involvement [16,24,25]. However, specific MRI findings (lesions in specific brain areas) are more likely to be associated with syndromic phenotypes, as in the case of MELAS (mitochondrial encephalomyopathy, lactic acidosis, and stroke-like episodes), Leigh, or Pearson/Kearns-Sayre syndromes [24,26,27]. Nonetheless, it should be stressed that brain MRI results can also appear normal in some patients with mitochondrial diseases.

Microcephaly, which was present in patients 1 and 2, has been described in both mitochondrial disorders and in ID cases [28], but its significance is poorly understood in terms of clinical presentation. Additionally, many patients with ID and mitochondrial disorders also have epilepsy [29] although, as reported here, EEG activity was abnormal in patient 3 and he had no clinical seizures, meaning that the lack of epilepsy does not exclude a mitochondrial disorder diagnosis. Some patients with mitochondrial diseases have hyperlactacidemia, which can be interpreted as a sign of mitochondrial metabolism impairment [30]. However, an increase in plasma lactate concentration is observable in many acute or chronic diseases and lactic acid in blood can be found within the normal range in some patients with mitochondrial disorders [2]. A persistent increase in plasma lactate concentration was found in all three cases and was accompanied by variable changes in pyruvate concentration (a slight increase in case 1, normal range in case 2, and a strong increase in case 3). Persistent hyperlactacidemia and an increased lactate/pyruvate ratio suggested to us that we should evaluate the presence of mitochondrial alterations by analyzing ETC activity in muscle tissue.

Our experience suggests that measuring the ratio of fasting lactate/pyruvate is recommendable in patients with ID with suspected mitochondrial disorders in order to further direct the diagnosis towards more relevant assays (*i.e.*, muscle biopsy, a detailed MRI/MRS study, or more sophisticated physiological measurements). In many cases a mitochondrial protein mutation is likely responsible for the clinical phenotype but in others, as reported here, no known mutations can be identified [31,32]. Based on these difficulties, and given that classical mitochondrial diseases affect only a small number of individuals with ID and ASD, we cannot exclude *de novo* mutations or secondary mitochondrial dysfunction in these patients. When these types of changes occur, they are accompanied by genetic abnormalities or biochemical defects in respiratory chain enzymes. Patient 1 and 3 had a 25%–50% complex II–IV deficiency, patient 1 also had slightly diminished levels of muscular CoQ10 (by 12%), and patient 2 displayed a 5%–15% decrease in complex III and IV activity. These biochemical findings are rare and add clinical relevance to this report since complex I deficiency is the most commonly observed deficit, and it presents either alone or in association with other respiratory complex deficits [33]. These cases adds new information compared to the previous reported cases of ASD and mitochondrial disease since the typical reported features such as gastro-intestinal symptoms (red-flags for, other organ involvement (eyes, liver, hearth, *etc*.) and regression were missing in these patients [21].

Pharmacological treatment for these patients remains limited, and a Cochrane Collaboration systematic review [34] concluded that there is currently no clear evidence supporting the systemic use of such interventions in patients with mitochondrial disorders. Symptomatic therapy can be effective in some patients with mitochondrial disorders [32,35] however a limited, or no, clinical effect is observed in the majority of cases [34]. It is clear that the efficacy of these treatments needs to be confirmed by randomized clinical trials performed in homogeneous study samples (with clinical features similar to the patients described in this report), which have clinically relevant primary endpoints. The limited size of our series limits our ability to recommend the use of any pharmacological treatments to treat patients with similar mitochondrial diseases. However, the treatments we administered to our patients could be described as almost empirical: there is currently very little evidence in the literature for drug combinations, which have a beneficial effect for such cases, and there are no FDA-approved drugs for this patient profile.

The administration of vitamins B1, B2, B3, folic acid (vitamin B9), carnitine, and Coenzyme Q, and their dosage was selected on the basis of the few reports we did find that show some benefit in patients with mitochondrial disease, as reviewed elsewhere [36,37]. Vitamin B6 and B12 were chosen because they have shown some benefit in patients with ASD and mitochondrial dysfunction [32,38]. Encouragingly, we observed a general (although variable) clinical improvement after pharmacological treatment with this combination, and interestingly, this beneficial effect was observed for ID (a condition which is classically considered to be stable over time and rarely scored after pharmacological treatment in this subgroup of patients), ASD, and in muscular tone signs. In contrast, other CNS manifestations such as sleep problems, irritability, and hyperactivity only improved following the administration of the neuroleptic drug risperidone. These results, although limited to case reports, suggest that administration of this mitochondrial “cocktail” as a symptomatic treatment can have some beneficial effects.

Undoubtedly the efficacy of this treatment should be evaluated in a large sample of patients with concomitant ID, ASD, hyperlactacidemia, and possible mitochondrial disorders. It is important to note that mitochondrial energy metabolism disorders may be missed in children with ID-ASD because its symptoms overlap with idiopathic ID, ASD, and other developmental disorders and secondary mitochondrial dysfunctions cannot be completely ruled out in these patients since their clinical phenotype fulfills that of a possible mitochondrial disorder [14]. In conclusion, our report supports the idea that some children with ID, ASD, and hyperlactacidemia may have a mitochondrial disorder with clinical manifestations different from, or less severe than, those patients with the known phenotypes of other well-characterized mitochondrial diseases.

These data offer clinicians a valuable set of benchmark parameters which can be used as a reference for suspected mitochondrial diseases in patients with ID, ASD, hypotonia, and for those with other non-systemic involvement (besides the muscular and nervous system). However it should be pointed out that these patients manifested additional and diverse clinical signs, *i.e.*, acquired microcephaly, hemi-spasticity, EEG alterations without clinical convulsions, and MRI imaging alterations, and non-specific pervasive developmental disorders (according to DMS-IV). The diagnosis of an associated mitochondrial disorder in patients with ID and ASD is sometimes difficult and requires knowledge of several different clinical presentations (as shown in Table 1). Each of these clinical features is shared by a subgroup of patients with idiopathic ID however these features alone are not sufficient to suggest mitochondrial disease unless other signs and symptoms are present. Despite the biochemical, histological, and genetic limitations that determine the final diagnosis of mitochondrial disease, this etiological possibility must be considered for primary or secondary mitochondrial respiratory chain disorders in children with ID and ASD in order to reach an early diagnosis and start prompt treatment and rehabilitation programs. There is a clear need of more advanced genetic testing of mtDNA and nDNA genes using next generation sequencing to help identify new genetic lesions (mutations) seen in these types of patients.

## 4. Experimental Section

### 4.1. Clinical Examinations

Each individual was subjected to a general physical, neurological, and psychological examination performed by a neuropediatrician, neurologist, and psychologist. Developmental delay was assessed under the age of 5 years using the standardized and validated Peabody Developmental Motor Scale 2 (PDMS-2) [39]. The Battelle developmental inventory (BDI-2) [40] was used to perform a comprehensive developmental assessment. The diagnosis of ID was made between 4.5–5 years of age using Raven’s Colored Progressive Matrices (CPM) [41]. This is a standardized instrument for assessing non-verbal general intelligence in children aged 5 years and over and for adults with ID for the purposes of clinical investigation. The overall score in each scale/inventory was transferred into a centile using the age-appropriate norms given by this scale. Testing was done in all three patients in order to exclude the common genetic causes of ID, such as fragile X syndrome, Prader–Willi syndrome, MECP2, and karyotype alterations (chromosomal deletions and duplications). Genetic testing for fragile X syndrome, Prader–Willi syndrome, MECP2 were done in University of Alabama (Med. Genomics Lab, Dr. L. Messiaen, Birmingham, USA) after extraction of genomic DNA from peripheral blood samples collected in EDTA (1 mM) in a volume of about 5 mL. Fragile X syndrome was analyzed by Southern blot analysis using primary restriction site EcoRI, (5.2 kb) and EagI (internal methyl-sensitive site) digestion, probed with StB12.3. After this analysis, fragile X syndrome was excluded in all three patients (CGG 29; 30; 48 for patient 1, 2 and 3, respectively). Analysis of MECP2 was performed by amplifying the MECP2-coding exons and flanking intronic sequences. The following primer pair was used to amplify exon 1: forward Rett-EX-1F 5'-GCAGCTCAATGGGGGCT-TTCAACTT-3' and reverse Rett-EX-1R 5'-GGCACAGTTA-TGTCTTTAGTCTTTGG-3'. PCR were performed and the products extracted from agarose gel using QIAquick gel extraction kit (Qiagen, Hilden, Germany) according to manufacturer’s protocol. Sequencing of the PCR products was performed by ABI prism BigDye Terminator (Applied Biosystem, Foster City, CA, USA) and separated by capillary electrophoresis and detected via laser induced florescence and compared with reference MECP2 sequence. Prader–Willi syndrome (PWS) was performed with the methylation test by Southern blot. For the analysis a double digestion was done with the enzymes Hind III and Hpa II (sensitive to methylation) and subsequent hybridization with the PW71B probe (patients without Prader–Willy or Angelman syndrome displayed a pattern with two bands (6 and 4 kb). Karyotype was performed at Oriente University, Faculty of Medicine in Ciudad Bolivar (Venezuela) by analyzing specimens that were processed using direct methods and unstimulated short-term (24- and 48-h) cultures with G-banding analysis (550–850 band). A minimum of 20 metaphases was required to define a normal karyotype. Analysis of known mitochondrial ASD/ID gene mutations according to patients’ phenotype are described in the Section 4.3. Autism spectrum disorder was assessed by Autism Diagnostic Interview—Revised (according to DMS IV criteria).

### 4.2. Blood Analysis

Blood samples were extracted in both the fasting and postprandial condition for analytical and biochemical assessments. In order to prevent erroneous lactate elevations due to a poor venipuncture technique or because of the use of a tourniquet, an indwelling butterfly needle was placed in order to permit blood sample collection after the patient had settled for 30 min.

### 4.3. Muscle Tissue Analysis

A quadricep muscle was biopsied and the sample was frozen until analysis with light and electron microscopy, as well as for mtDNA content using real time PCR analysis and for the following mutations: T3271C and A3243G in tRNA-leu (UUR), A8344G and T8356C in tRNA-lys, T8993C and T8993G in subunit 6 of ATPase gene, G3460A and G11778A, mutation at 8993 and common deletion of mitochondrial DNA that causes Kearns-Sayre syndrome (KSS), progressive external ophthalmoplegia (PEO) or Pearson syndrome. MtDNA deletions and depletion were analyzed as previously described [42,43]. The percentage of m.6955G>A transition was analyzed by last-cycle radioactive PCR/RFLP by using primers HmtL6954 (GGATTCATCTTTCTTTTCACCCTAG) and HmtH7114 (TGGCGTAGGTTTGGTCTAGG). HmtL6954 contains a mismatch (G-C) at nucleotide position 6951. The amplicon size is 204 bp and the PCR conditions: 94 °C 2min (94 °C 30 s/55 °C 30 s/72 °C 1 min 30 s) 35 cycles, 72 °C 5 min. 0.5 μL of (α-32P) dCTP at 10 mCi/mL was added to each PCR reaction before the last cycle. The restriction enzyme BlnI (C/CTAGG) cuts the amplicon in two fragments of 183 + 21 bp. The m.6955G>A transition removes the cutting site. The levels of the mutant mtDNA were quantified by using the GelProAnalyzer 4.0 program. The revised human mitochondrial DNA Cambridge reference sequence (GenBank REFSEQ AC_000021.2) was used. All of these mtDNA depletion analyses were negative.

### 4.4. Determination of Mitochondrial Respiratory Chain Complexes

Small pieces of frozen muscle tissues were homogenized (1/30 weight per volume) in a solution containing 50 mM Tris buffer (pH 7.5), 100 mM potassium chloride, 5 mM MgCl_2_, and 1 mM ethylenediaminetetraacetic acid using a glass/glass homogenizer. Enzyme activities were assayed at 30 °C using a spectrophotometer and were calibrated against CS activity.

ETC function analysis is presented as enzyme complex activity, namely: Nicotinamide adenine dinucleotide (NADH): Coenzyme Q1-oxidoreductase (complex I), NADH: Cytochrome c-oxidoreductase (complex I + III), succinate: Cytochrome c-oxidoreductase (complex II + III), ubiquinone: Cytochrome c-oxidoreductase (complex III), cytochrome c-oxidase (complex IV), in all cases using CS as a mitochondrial marker enzyme, as previously described [44,45]. The concentration of CoQ10 was determined according to Montero *et al.* [46]. Abnormalities in ETC function were compared to the standard range in age-matched control subjects used in each diagnostic laboratory.

Each patient’s parents were requested (and gave) written informed for consent to allow their child’s medical information to be anonymously abstracted into a clinical database that contained their medical history, physical examination findings, and the results of neurological, psychological, and metabolic tests. The protocols described in this manuscript follow rules approved by the institute’s ethical committee.

## 5. Conclusions

In conclusion, our experience suggests that children with ID, ASD, and with abnormal neurological or muscular involvement but without other systemic findings should be also evaluated for mitochondrial disorders (blood biochemical screening tests) and these disorders should be included in the differential diagnosis of secondary ID/ASD. Other genetic causes such as single gene disorders with ID or ASD as a feature via candidate gene mutation screening, clinical genetics evaluation and microarray analysis should be performed in patients presenting with these findings. Advanced genetic testing results could impact on clinical care and genetic risk counseling for family members.
